# Optimized Protein Extraction for Quantitative Proteomics of Yeasts

**DOI:** 10.1371/journal.pone.0001078

**Published:** 2007-10-24

**Authors:** Tobias von der Haar

**Affiliations:** Protein Science Group, Department of Biosciences, University of Kent, Canterbury, United Kingdom; Victor Chang Cardiac Research Institute, Australia

## Abstract

**Background:**

The absolute quantification of intracellular protein levels is technically demanding, but has recently become more prominent because novel approaches like systems biology and metabolic control analysis require knowledge of these parameters. Current protocols for the extraction of proteins from yeast cells are likely to introduce artifacts into quantification procedures because of incomplete or selective extraction.

**Principal Findings:**

We have developed a novel procedure for protein extraction from *S. cerevisiae* based on chemical lysis and simultaneous solubilization in SDS and urea, which can extract the great majority of proteins to apparent completeness. The procedure can be used for different *Saccharomycetes* yeast species and varying growth conditions, is suitable for high-throughput extraction in a 96-well format, and the resulting extracts can easily be post-processed for use in non-SDS compatible procedures like 2D gel electrophoresis.

**Conclusions:**

An improved method for quantitative protein extraction has been developed that removes some of the sources of artefacts in quantitative proteomics experiments, while at the same time allowing novel types of applications.

## Introduction

The recent literature has seen a significant increase in the number of publications that attempt the determination of protein abundances in yeast cells on a large scale [1]–[4]. These studies provide an important data source for the emerging fields of systems biology and control analysis, where macromolecular abundance data are required for the construction of meaningful models. However, a detailed comparison showed that correlations between data sets generated by different groups are generally poor (see the supplementary data in Lu *et al.,* ref. 3). A good illustration of the variability of published abundance data is given by the example of translation elongation factor eEF2, for which values during logarithmic growth in YPD at 30°C are given as 78,100; 321,782; and 8,764 proteins per cell [2]–[4]. Importantly, this spread of reported abundance values is representative for the data set as a whole, since the weighted standard deviation for reported eEF2 abundance equals the median of weighted standard deviations for data sets of all individual proteins (TvdH, unpublished).

With the exception of one study [4], all of the work cited above analyzed protein abundance following the extraction of these molecules from cells. Importantly, none of these studies evaluated the efficiencies of the respective extraction procedures they employed. During attempts to quantify intracellular levels of the polypeptide release factors eRF1 (Sup45p) and eRF3 (Sup35p) in *S. cerevisiae*, we found that apparent abundance values for both factors varied widely depending on the exact protein preparation procedures used. Although the poor consistency between genome-wide protein abundance studies is likely to arise from many different sources, these observations suggest that varying extraction efficiencies could be one factor contributing to high data variance.

Our problems with the quantification of release factor levels prompted an attempt to develop an improved, more quantitative protein extraction procedure. Criteria for an ideal approach were a), quantitative extraction and solubilization of all *S. cerevisiae* proteins, b) maintenance of the proteome in the pre-extraction state, c) easy quantification of the numbers of extracted cells to aid in the determination of absolute protein levels per cell, and d) a minimum of manual intervention in order to make the procedure easily applicable and amenable to high-throughput experimental approaches.

## Results

### Basic Procedure

As starting point for the development of an improved method, we chose a published “alkaline lysis” procedure [5], which in our hands gave the highest extraction efficiency of the different approaches initially tested (data not shown). In the original protocol, yeast cells are harvested, resuspended in 0.1 N NaOH and incubated for several minutes, harvested again and then resuspended and boiled in standard SDS-PAGE sample buffer. Although the exact mode of cell lysis is not clearly understood, it appears to be the combined action of NaOH in the pre-lysis buffer and of 2-mercaptoethanol in the sample buffer that makes cell walls porous enough for proteins to escape into the surrounding buffer. The initial treatment with NaOH leads to some membrane damage, since small molecules are readily released during this incubation. In contrast, bulk protein is only released once the cells are boiled in sample buffer. It should be noted that cell walls are not completely destroyed during the extraction, since the cells remain visible as “ghosts” throughout the entire procedure. The same is also true for the modified procedure described below.

Although generally of high efficiency, this procedure has drawbacks for the purposes of accurate protein quantification. Small proteins (<15 kDa) are released during the NaOH incubation, and are therefore underrepresented in the final extract. Second, yeast cells remain viable during the several minutes of NaOH incubation [5]. Cells may therefore respond to this harsh treatment with significant proteome alterations, and the final extract may not reflect the proteome composition under normal culture conditions. Lastly, although yeast cell density can be accurately quantified during the NaOH incubation step, subsequent centrifugation and resuspension steps often lead to a partial loss of cells which is difficult to control. Once resuspended in sample buffer, accurate cell quantification is made difficult by the presence of the loading dye.

In order to circumvent these problems, the basic procedure from reference 5 was initially modified as follows. Harvested cells were resuspended in a solution containing NaOH, 2-mercaptoethanol and SDS and immediately heated to 90°C, thus achieving simultaneous lysis and solubilization. In the next step, the extract was neutralized, and then Tris-HCl buffer, glycerol and bromophenol blue were added to make up a final buffer composition very similar to standard SDS-PAGE sample buffer. This extract could immediately be loaded onto SDS-PAGE gels or further processed for non-SDS compatible applications (see below).

### Optimization of total protein extraction

As an initial control experiment, it was tested how stable proteins were under the hot alkaline buffer conditions of the first extraction step. Incubation of a yeast extract under these conditions did not detectably change the pattern of protein bands for up to 20 minutes, although the band pattern did degrade upon prolonged incubation (>1 hour, data not shown). In contrast, once the NaOH in this buffer was neutralized, the protein band pattern was stable almost indefinitely at this temperature. The lysis step was therefore limited to a ten minute incubation under alkaline high temperature conditions, in order to ensure that the proteome was not artifactually altered during the lysis procedure.

**Figure 1:**
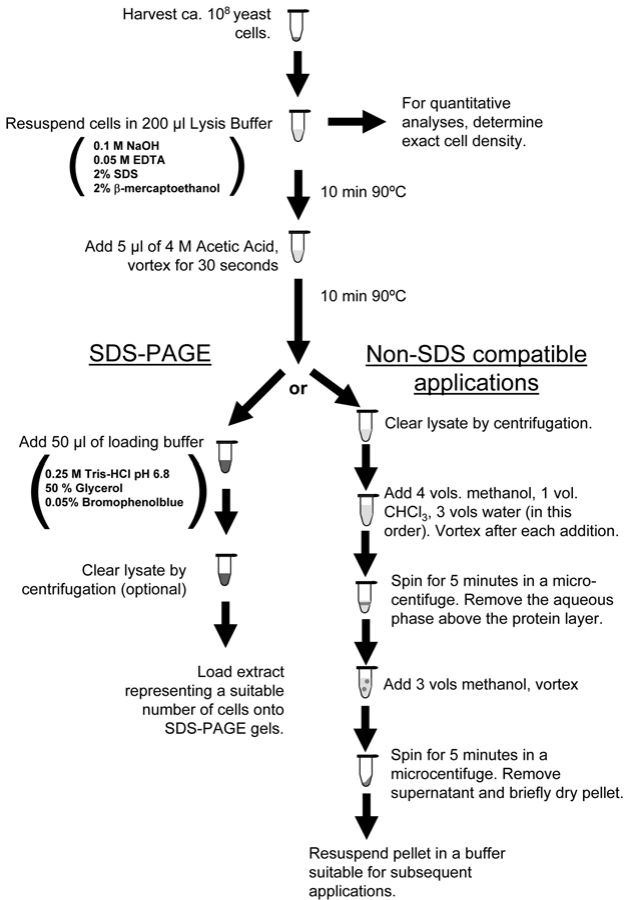
Outline of the basic extraction procedure. Yeast cells are harvested, resuspended in lysis buffer 1, and heated to 90°C for ten minutes. The lysate is then brought to neutral pH, and heated again to increase solubilization. Following this step, the lysate can either be brought to a composition similar to standard SDS-PAGE sample buffer and immediately used in gel electrophoresis-based applications (left branch), or the proteins can be precipitated and the SDS-containing buffer can be removed for SDS-incompatible applications (right branch).

The procedure was then optimized by systematic variation of parameters like SDS concentration, boiling time, and inclusion of additional solubilizing agents. Extraction in these experiments was assessed by determining the amount of total protein that could be released from a fixed number of cells. The optimized procedure, which among all the conditions tested extracted the maximum amount of protein (corresponding to ∼5×10^−12^ g protein per cell), is given in Figure 1.

**Figure 2:**
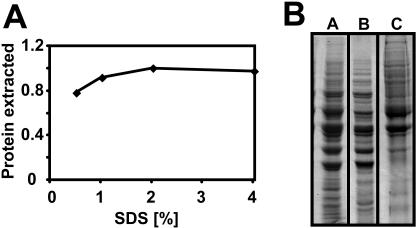
Extraction efficiency is limited by the ratio of SDS to cells. (A) The amount of total protein that can be extracted at a density of 5×10^8^ cells/ml is shown as a function of SDS-concentration in the lysis buffer. Other buffer components were kept constant (0.1 M NaOH, 0.05 M EDTA, 2% 2-mercaptoethanol). SDS-concentrations below ∼2% limit extraction efficiency at this cell concentration. (B) Cells were lysed in buffer containing 2% SDS, at cell densities of 5×10^8^ cells/ml (Lane A) or 2.5×10^9^ cells/ml (Lane B). Extract representing 5×10^6^ cells was resolved on a 10% SDS-PAGE gel. The lower intensity of many bands in lane B shows that extraction efficiency is limited at the higher cell density. Lane C shows a re-extraction of the cells from lane B at a cell density of 5×10^8^cells/ml. In this lane, twice the amount of extract (10^7^ cells) was loaded compared to lanes A and B in order to make bands that were under-extracted in sample B more clearly visible.

In these experiments, the single most important parameter for efficient extraction was the ratio of SDS to cells during the hot alkaline incubation in lysis buffer (Figure 2). The conditions given in figure 1 correspond to 5×10^8^ cells/ml in a solution containing 2% SDS. If this ratio was altered, either by decreasing the SDS concentration (Figure 2A) or by increasing the cell density (Figure 2B), extraction quickly became sub-optimal. Importantly, this reduction in extraction efficiency did not affect the proteome uniformly. The gel shown in figure 2B demonstrates a bias against extraction of larger proteins when the cell density was increased (compare lane A, extracted at 5×10^8^cells/ml, versus lane B, extracted at 2.5×10^9^ cells/ml). These proteins remained associated with the cellular debris and could be recovered by a second extraction, as shown in lane C of this gel (this sample is two-fold concentrated compared to lanes A and B). In contrast, at the “correct” SDS-to-cell ratio, no protein could be recovered in a second extraction step (data not shown). Thus, the procedure shown in figure 1 achieved maximal release of total protein into the extraction buffer, without producing apparent alterations to the proteome.

**Figure 3:**
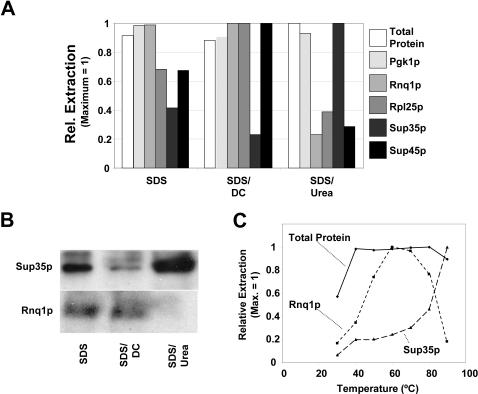
A subset of proteins is sensitive to additional solubilizing factors. (A) Protein extracts were prepared in the absence or presence of 1% deoxycholate (DC) or 8 M Urea. The amount of extracted total protein and individual proteins was then determined using gel electrophoresis and western blotting. Rpl25p and Sup45p show an increase in extraction efficiency in the presence of DC, whereas Sup35p shows a decrease. Conversely, Sup35p is more efficiently extracted in the presence of Urea, while Rnq1p, Rpl25p and Sup45p are extracted less efficiently. (B) shows the western blot for the data presented in panel A for Sup35p and Rnq1p. (C) Temperature dependence of extraction in the presence of Urea for total protein, Rnq1p and Sup35p. Rnq1p is extracted to maximum efficiency at ∼60°C, but decays at higher temperatures, whereas Sup35p requires high temperatures for efficient extraction.

Western blotting experiments showed that many individual proteins are extracted to maximum efficiency with the conditions given in figure 1, consistent with the data obtained for total protein (Act1p, Hsp104p, Pgk1p, Yef3p, Sui2p, Rnq1p, Rpp0; figure 3B and data not shown). Surprisingly however, a minority of protein species showed extraction patterns that were significantly different. In particular, inclusion of extra solubilizing agents significantly affected the extraction efficiency for Sup35p, Sup45p, and Rpl25p (figure 3B and C). The inclusion of 1% deoxycholate significantly increased extraction efficiencies for Sup45p and Rpl25p, but it also significantly decreased extraction efficiencies for Sup35p. The latter protein was extracted with greatly increased efficiency when 8 M Urea was included in buffer 1, but under these conditions the abundance of many other proteins in the extracts dropped dramatically.

The most straightforward interpretation of these results is that conditions as shown in Figure 1 are sufficient for the maximal extraction of the great majority of proteins, but that some proteins require harsher conditions for efficient transfer to the solubilized state. Surprisingly, however, these data also show that conditions which increase solubilization of one protein species decrease the abundance of other proteins in the extract. In the case of deoxycholate, the molecular mechanisms underlying the decrease in Sup35p extraction are not clear. In contrast, in the case of urea, it is likely that the apparently lower extraction efficiencies for many proteins are not actually the result of reduced extraction, but rather of degradation of these proteins.

Urea in solution establishes an equilibrium with cyanate, which covalently modifies several different amino acid side chains in a reaction that is greatly accelerated by heat and alkaline conditions [6]. These modifications may lead to a destabilization of some proteins under the extraction conditions. In order to test whether a reduction in incubation temperatures might prevent destabilization of apparently urea-sensitive proteins but still permit efficient solubilization of Sup35p, extraction with the urea containing buffer was attempted over a range of incubation temperatures (figure 3D). Urea sensitive proteins like Rnq1p were indeed stabilized by lowering the extraction temperature. However, efficient Sup35p extraction required raising the temperature to a point where Rnq1p levels became nearly undetectable.

The example of Sup35p and Rnq1p illustrates the difficulty in finding extraction conditions that are equally efficient for all cellular proteins. It should be noted, however, that proteins which can not be extracted to apparent completeness in simple SDS buffer are the exception rather than the rule. Thus, none of the proteins represented by bands visible in gels stained for total protein increased significantly when deoxycholate or Urea were included in the extraction buffer.

### Analysis of non-extracted proteins

The previous experiments analyzed extraction efficiency in terms of the maximum amount of protein recovered from a certain number of cells. However, populations of some proteins may be very tightly associated with the post-extraction cellular debris, and may thus not be extractable at all. Proteins remaining associated with the cellular structures following extraction were therefore analyzed by two independent means.

**Figure 4:**
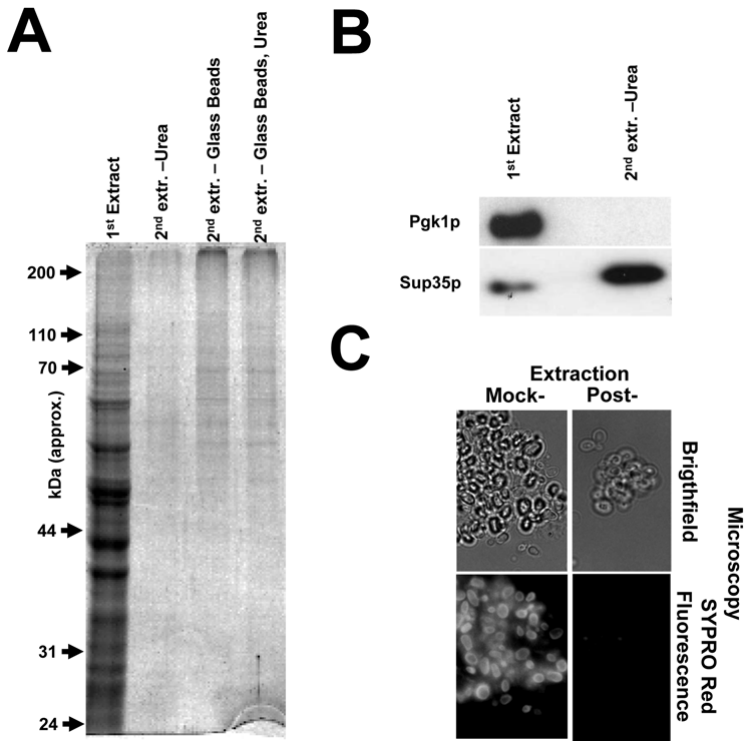
Extraction is near complete for the majority of proteins. (A) Yeast cells were extracted in a first round with the procedure described in figure 1 and recovered. They were then subjected to a second round of extraction either by boiling in sample buffer also containing 8 M Urea (sample “Urea”), by glass bead lysis followed by boiling in normal sample buffer (sample “Glass Beads”), or by glass bead lysis followed by boiling in sample buffer also containing 8 M Urea (sample “Glass Beads, Urea”). Note that the second extracts are twice concentrated with respect to the first extract. (B) Western blotting analysis of samples from panel A. The western blot using anti-Sup35 antibodies confirms that this particular protein requires Urea for efficient extraction. In contrast, other individually tested proteins were not recovered by a second Urea-based extraction (Pgk1p is shown here as a representative example). (C) The total protein content of mock-extracted and extracted yeast cells was visualized by staining with the fluorescent protein stain Sypro Red. The Sypro Red Signal drops by >95% following application of the extraction procedure.

First, samples of cells were subjected to the new procedure, collected by centrifugation, and then extracted a second time using a variety of alternative procedures. Re-extraction by alkaline lysis in the presence of 8 M urea did not yield significant extra bands in an SDS-PAGE gel (figure 4A). This confirms that protein species that are only extractable with urea (such as Sup35p, figure 4B) constitute a minority among yeast proteins. Some extra proteins appeared to be liberated by physical breakage of the cell “ghosts” using glass beads, although these constitute a clear minority among yeast protein species. Note that the re-extracted samples in figure 4 are twice concentrated compared to the first extraction sample.

As a second approach to analyzing potential non-extractable proteins, cells that had been extracted as described in figure 1 were stained with a fluorescent total protein stain (SYPRO Red), and analyzed using fluorescence microscopy. These samples were compared to mock-extracted cells, which had been treated with 0.1 N NaOH for ten minutes, a treatment that does not release any proteins larger than ∼15 kDa from the cells. Figure 4C demonstrates that mock-extracted cells showed bright fluorescence consistent with a high protein content, whereas the extracted cells showed very low levels of fluorescence near the detection limit. On average, the fluorescent signal per cell dropped by >95%, again confirming that the great majority of yeast proteins are quantitatively extracted by the alkaline lysis procedure.

### Extraction from different growth conditions and of other yeast species

in order to test the applicability of the new procedure, *S. cerevisiae* cells recovered from a variety of growth conditions were extracted. Extraction was efficient for cells growing in liquid as well as on plates, and also for stationary phase cells. Moreover, tests with other yeast species showed that this procedure worked well with other yeasts from the *Saccharomycetes* group including *S. bayanus, Candida albicans* and *Pichia pastoris*.

### High-throughput extraction

**Figure 5:**
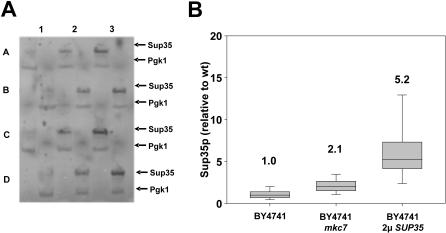
Yeast Protein extraction in 96 well format. Different yeast strains were grown in a 96 well plate format, followed by protein extraction and gel electrophoretic analysis in the same format. (A) Shows the western blot for a 3×4 well section from the 96 well plate. Pgk1p and Sup35p were detected simultaneously by mixing the respective antibodies. Note that the 96 well electrophoresis system employed here uses a staggered row format, hence the change in alignment between the different rows. The three columns correspond to three strains with different Sup35p expression levels (see text for further explanation). (B) Box plot summarizing variability of the Sup35p abundance data generated from all 24 wells for each strain. Sup35p levels were normalized to Pgk1 levels. Indicated are the mean (central line in the boxes and numbers above the boxes), 25^th^/75^th^ percentiles (box limits), and 10^th^/90^th^ percentiles (feathers).

The new extraction procedure is also well suited to application in a high-throughput context. Yeast cells can be easily processed in 96 well plates, and this format is sufficiently sensitive e.g. for the identification of strains that show altered expression levels of a particular gene product. Three yeast strains showing different expression levels of Sup35p were employed in a pilot experiment: BY4741, which contains wild-type levels of this protein, the same strain containing a deletion for the *mkc7* gene, which as we previously observed shows moderately increased levels of Sup35p (TvdH and M.F. Tuite, unpublished), and BY4741 transformed with the *SUP35* gene on a high copy plasmid, which shows the highest levels of Sup35p. These strains were grown in 24 independent micro-cultures each in the wells of a 96 well plate, harvested and extracted with minor modifications to the procedure described in Figure 1 (a detailed description of the 96-well extraction is given in the Materials and Methods section). The resulting protein samples were separated on a 96-well gel electrophoresis system, transferred onto a nitrocellulose membrane, and Sup35p and Pgk1p in the samples were detected by western blotting using a mixture of the two respective primary antibodies (Pgk1p levels were used as an internal loading control). Figure 5A shows a 3×4 well section of the resulting blot, containing four independent samples for each of the three strains.

Importantly, the different strains showed significantly different growth rates and final cell densities, thus mimicking conditions which would be encountered in a real-life screening experiment involving many different strains. These differences in growth were efficiently corrected for by use of the internal loading control. Figure 5B shows that this procedure could reliably differentiate between the different Sup35p expression levels in the three strains. From the spread of the data for each strain, it can be estimated that a moderate two-fold change in expression level can be detected with ≥80% confidence in individual experiments. As an alternative to the use of an internal loading control, the optical density of the 96-well cultures can be determined, however, this increases the variability in the data, presumably because it does not correct for different rates of recovery of cells during harvesting.

### Post-processing for non-SDS compatible applications

**Figure 6:**
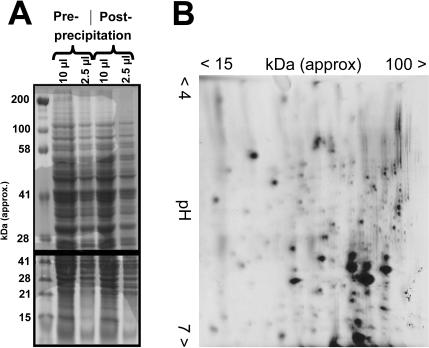
Post-processing of samples for non-SDS compatible applications. (A) A protein extract was prepared, split in half and one half directly applied to an SDS-PAGE gel. The other half was methanol-precipitated, resuspended in the same volume of SDS-PAGE sample buffer as the non-precipitated sample, and then applied to the same gel. The identical band patterns for the two samples indicate that samples can be precipitated and resuspended without any apparent loss of proteins, with the exception of very large proteins (>150 kDa) which appear to be partially lost or degraded during the precipitation procedure. (B) 25 µg of precipitated protein (representing ∼1×10^7^ cells) were subjected to two-dimensional gel electrophoresis in order to demonstrate the suitability of this procedure for efficient SDS removal.

A drawback of the alkaline/SDS extraction procedure is the presence of SDS in the final extracts, which precludes its application for many important proteomic techniques that are not SDS-compatible. However, methanol-induced precipitation of proteins can be efficiently used for removal of SDS from protein extracts (for more details, see reference 7 and the right hand branch of the flow chart in figure 1). Figure 6A shows the result of an experiment where protein from one half of an SDS-containing extract was precipitated, resuspended in sample buffer, and applied next to an aliquot of the non-precipitated portion of the sample. The banding pattern and overall band intensity are indistinguishable for the original and precipitated samples in the molecular weight range between ∼10 and ∼150 kDa, thus demonstrating that precipitation by this method is highly quantitative and does not change the apparent composition of the sample. The only exception are very large proteins (>150 kDa), for which partial loss or degradation occur during the precipitation procedure. Figure 6B shows that the precipitation removes the SDS sufficiently for analysis using non-SDS compatible two-dimensional gel systems.

## Discussion

A new alkaline/SDS extraction procedure has been developed that can quantitatively solubilize and extract the large majority of yeast proteins. This is demonstrated by observations that the apparent extraction efficiency for total protein could not be increased when extraction conditions were systematically altered, that re-extraction of the cell pellets with alternative methods yielded very few additional bands, and that direct probing for protein remaining in the cells using a fluorescent protein stain showed a drop in fluorescence by more than 95 per cent following extraction.

Our data also clearly show that extraction of individual proteins can proceed in ways that are very different from extraction of the bulk proteome. Despite many attempts to alter the conditions in ways that would allow quantitative extraction of all proteins, the solubilization of some proteins always required conditions that destabilized others. For quantifying individual proteins, a routine starting point should therefore be the assessment of different extraction procedures, including different solubilizing agents and mechanical disruption. However, proteins that are not maximally extracted when using SDS as sole solubilizing agent represent a minority of protein species. For simultaneous large scale (i.e., proteome-wide) quantifications, extraction using these conditions will therefore yield the most accurate results for the majority of proteins.

The molecular causes underlying poor extraction for some proteins remain to be established. The fact that cell wall structures remain intact during the extraction procedure (Figure 4C) suggests that proteins must diffuse across them during extraction. Since physical disruption of the remaining cell wall structures generates a few additional bands on SDS-PAGE gels (figure 4A), this diffusion process may be limiting for some gene products. However, extraction of much larger proteins than the ones liberated by physical breakage proceeds efficiently using alkaline extraction only (figure 4A), so that these species must constitute some special case in terms of their extraction requirements.

It should be noted that there are two known types of covalently cell-wall associated proteins in *S. cerevisiae*, namely those linked to the β-1,3-glucan matrix through an unknown alkali-sensitive linkage, and those linked to the same matrix in a glycosylphosphatidylinositol- (GPI-) dependent manner [8]. Of these, the former should be readily released during the alkaline incubation step, whereas the latter can normally only be released from cell walls by enzymatic destruction of the covalent linkage. It is therefore unlikely that the protein species liberated after physical breakage correspond to conventional cell wall proteins.

Especially difficult to understand is the varying extraction efficiency observed for Rpl25p, Sup45p and Sup35p. All three proteins are presumed to be soluble and to show a cytoplasmic localization [9]. Rpl25p is a large ribosomal subunit protein that is not known to be tightly associated with any cellular structures, and Rpp0p, another protein component of the large ribosomal subunit, is efficiently extracted using only SDS as solubilizing agent. One of the differences between these two proteins is that Rps25p makes strong direct contacts with the ribosomal RNA and is part of the large ribosomal subunit core [10], whereas Rpp0 is more peripherally associated with this complex [11]. It may thus be the detachment from a tightly associated rRNA-protein complex that limits solubilization of Rps25p and that requires deoxycholate as additional detergent.

Sup45p and Sup35p are translation termination factors that act on ribosomes containing stop codons in their A-site, catalyzing release of the nascent polypeptide [12]. Sup35p can exist in an aggregated prion form in *S. cerevisiae* [13], however, the strain used in this study has no such prion-type aggregates (data not shown). Moreover, although prion aggregates are resistant to treatment with SDS at room temperature, they can normally be disrupted by boiling in 2% SDS [14]. Nevertheless, the fact that additional Sup35p was released from SDS-extracted cells by re-solubilizing in urea, but not by physically destroying the cell wall structures and re-solubilizing in the absence of urea, suggests that the problem with Sup35p extraction lies in the solubilization of this protein, rather than in liberation of the protein from the cell structures once solubilization has occurred.

In conclusion, this study describes a novel protein extraction procedure for *Saccharomycetes* yeasts that combines several desirable aspects. Proteins are denatured very rapidly following cell harvest, thus minimizing proteome alterations during the extraction procedure. The majority of proteins are quantitatively solubilized, and the number of extracted cells can be determined very accurately, thus forming an ideal basis for the absolute quantification of protein content per cell. Samples are easily post-processed for non-SDS compatible applications, thus making the procedure widely applicable. Lastly, the fact that relatively little manual intervention is required for the preparation of extracts allowed us to develop a novel high-throughput version of this procedure.

## Materials and Methods

### Yeast strains and growth conditions

*S. cerevisiae* BY4741 (MAT**a** *leu2-Δ0 met15-Δ0 ura3-Δ0 his3-Δ0*)[15] was grown in liquid rich medium (2% glucose, 1% peptone, 1% yeast extract) and harvested from logarithmically growing cultures (oD_600_≤1) except where otherwise stated.

### Preparation of yeast extracts

The basic extraction procedure is described in detail in the Results section and in figure 1. For the re-extraction experiments, cells that had been subjected to a first extraction procedure were pelleted, washed briefly with 200 µl of 0.1% SDS, and re-extracted as described. For mechanical disruption, cells were resuspended in 200 µl of SDS sample buffer. 1 volume of glass beads was added, and the samples beaten in a Precellys 24 bead beater (Stretton Scientific Ltd., 3 cycles of 30 seconds beating, 30 seconds pause).

*Gel electrophoresis and western blotting* were carried out using standard procedures [16], [17]. Protein gels were incubated in SYPRO Red stain (Invitrogen) and visualized using an FLA5200 laser scanner (Fuji). Western blots were performed using FITC-labeled secondary antibodies (Sigma Aldrich), and were scanned on the same instrument.

### Microscopy

Cells were pelleted, washed briefly in 200 µl of 0.1% SDS and incubated in 1 ml of 5% formaldehyde at room temperature for 30 minutes. Cells were then resuspended in 1 ml of 0.01% SDS containing SYPRO Red stain at a dilution of 1∶5000, and observed under a fluorescence microscope.

### Extraction in 96-well plate format

Wells of a 96 well deep-well plate (2 ml well capacity, Whatman) were filled with 600 µl of medium and inoculated with the different yeast strains. Following overnight growth, 50 µl of culture per well were transferred to a fresh microtitre plate, and the oD_595_ was determined using a 96 well plate reader. 250 µl of culture per well were then transferred to a 96 well microfilter plate (0.45 µ cellulose acetate, Whatman), and centrifuged at 2500 g for 5 minutes. The filtrate was discarded, and the last step was repeated once with 250 µl of additional culture to bring the total volume of harvested culture to 500 µl. For lysis, yeast cells were recovered from the filter surfaces by resuspension in 45 µl of buffer 1 lacking SDS but also containing 8 M Urea (i.e. 8 M Urea, 0.1 N NaOH, 0.05 M EDTA, 2% 2-mercaptoethanol). SDS was omitted from this buffer and only added in the subsequent step because excessive foaming was observed when trying to resuspend the cells in its presence. 8 M Urea was included because we wanted to determine the levels of Sup35p in the extracts, which requires this compound for efficient solubilization (see the results section for details).

Resuspended cells were mixed with 5 µl of 20% SDS in the wells of a 96-well PCR plate, and heated to 90°C for 10 minutes in a thermocycler. The uncleared lysates were directly transferred to the wells of a 96-well protein electrophoresis gel (E-PAGE *96*, Invitrogen), resolved and transferred onto nitrocellulose membrane using a dry blotting format (iBlot, Invitrogen). Detection of proteins was performed as for mini-gel based western blots.
